# Supplementary material for: In Vitro and In Silico Analyses of New Cinnamid and Rosmarinic Acid-Derived Compounds Biosynthesized in Escherichia coli as Leishmania amazonensis Arginase Inhibitors
Source: Pathogens. 2022 Sep 7;11(9):1020. doi: 10.3390/pathogens11091020 (PMC9504950; doi:10.3390/pathogens11091020)
Supplement: Supplementary file 1 [file pathogens-11-01020-s001.zip › pathogens-1885600-supplementary.pdf]

*pathogens*

*Supplementary Materials*

# In Vitro and In Silico Analyses of New Cinnamid and Rosmarinic Acid-Derived Compounds Biosynthesized in *Escherichia coli* as *Leishmania amazonensis* Arginase Inhibitors

**Julio Abel Alfredo dos Santos Simone Come [1,†], Yibin Zhuang [2,3,†], Tianzhen Li [2,3], Simone Brogi [4,*], Sandra Gemma [5], Tao Liu [2,3,*] and Edson Roberto da Silva [6,*]**

1 Departamento de Pré-Clínicas, Faculdade de Veterinária, Universidade Eduardo Mondlane, Av. de Moçambique, Km 1.5, Maputo CP 257, Mozambique
2 Tianjin Institute of Industrial Biotechnology, Chinese Academy of Sciences, Tianjin 300308, China
3 Key Laboratory of Systems Microbial Biotechnology, Chinese Academy of Sciences, Tianjin 300308, China
4 Department of Pharmacy, University of Pisa, Via Bonanno 6, 56126 Pisa, Italy
5 Department of Biotechnology, Chemistry and Pharmacy, University of Siena, Via Aldo Moro 2, 53100 Siena, Italy
6 Laboratório Farmacologia e Bioquímica (LFBq), Departamento de Medicina Veterinária, Universidade de São Paulo Faculdade de Zootecnia e Engenharia de Alimentos, Pirassununga 13635-900, SP, Brazil
* Correspondence: simone.brogi@unipi.it (S.B.); liu_t@tib.cas.cn (T.L.); edsilva@usp.br (E.R.d.S.); Tel.: +39-050-2219613 (S.B.); +86-22-24828718 (T.L.); +55-19-35656828 (E.R.d.S.)
† These authors contributed equally to this work.

**Citation:** Come, J.A.A.d.S.S.; Zhuang, Y.; Li, T.; Brogi, S.; Gemma, S.; Liu, T.; da Silva, E.R. In Vitro and In Silico Analyses of New Cinnamid and Rosmarinic Acid-Derived Compounds Biosynthesized in *Escherichia coli* as *Leishmania amazonensis* Arginase Inhibitors. *Pathogens* **2022**, *11*, 1020. https://doi.org/10.3390/pathogens11091020

Academic Editor: Nicola Carter

Received: 10 August 2022
Accepted: 6 September 2022
Published: 7 September 2022

**Publisher's Note:** MDPI stays neutral with regard to jurisdictional claims in published maps and institutional affiliations.

**Copyright:** © 2022 by the authors. Licensee MDPI, Basel, Switzerland. This article is an open access article distributed under the terms and conditions of the Creative Commons Attribution (CC BY) license (http://creativecommons.org/licenses/by/4.0/).

## Table of Contents

**Figure S1** $^{1}H$ spectrum of isolated compound **8** in $CD_3OD$ **Page S2**
**Figure S2** $^{13}C$ spectrum of isolated compound **8** in $CD_3OD$ **Page S2**
**Figure S3** $^{1}H$ spectrum of isolated compound **9** in $CD_3OD$ **Page S3**
**Figure S4** $^{13}C$ spectrum of isolated compound **9** in $CD_3OD$ **Page S3**
**Figure S5** $^{1}H$ spectrum of isolated compound **10** in $CD_3OD$ **Page S4**
**Figure S6** $^{13}C$ spectrum of isolated compound **10** in $CD_3OD$ **Page S4**
**Figure S7** MS spectra of compounds **8-10** **Page S5**

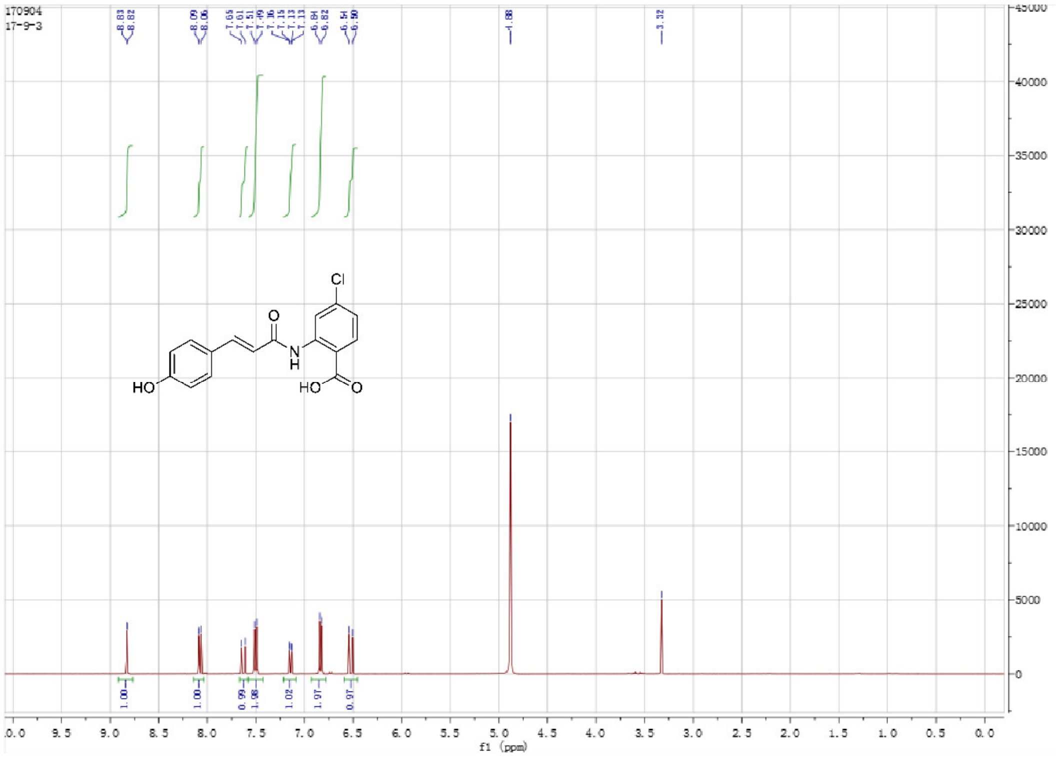


**Figure S1.** $^{1}H$ spectrum of isolated compound **8** in $CD_3OD$.

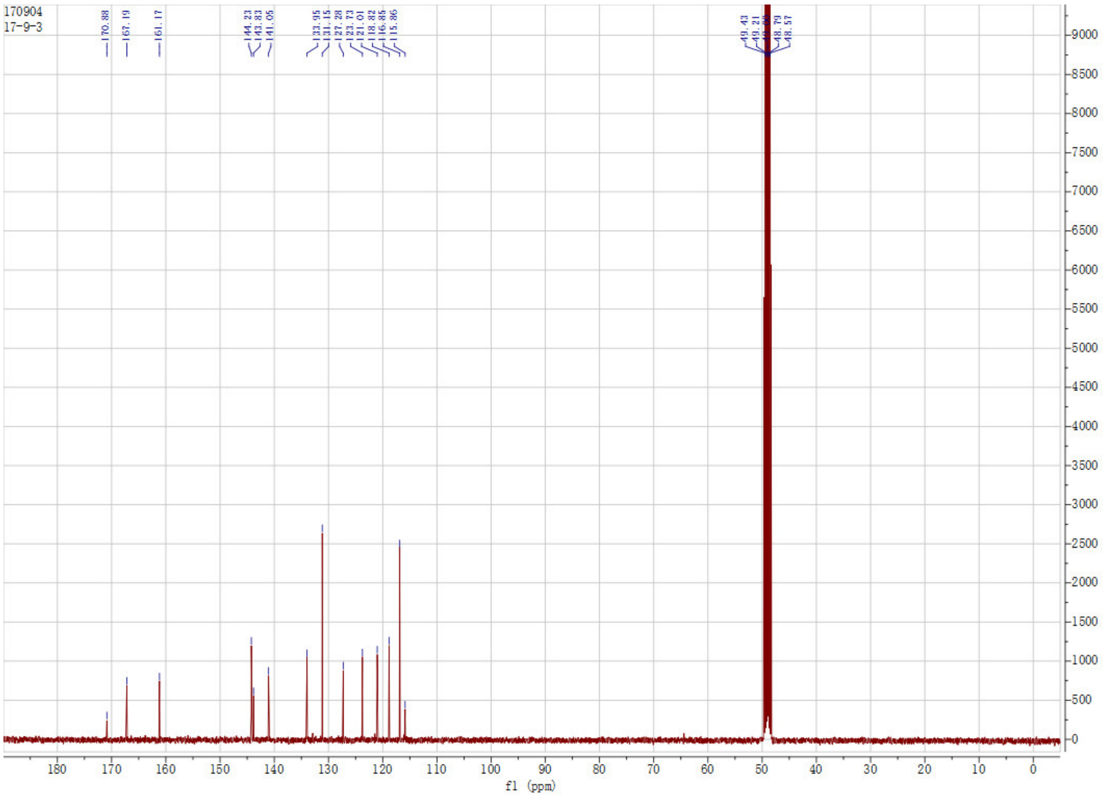


**Figure S2.** $^{13}C$ spectrum of isolated compound **8** in $CD_3OD$.

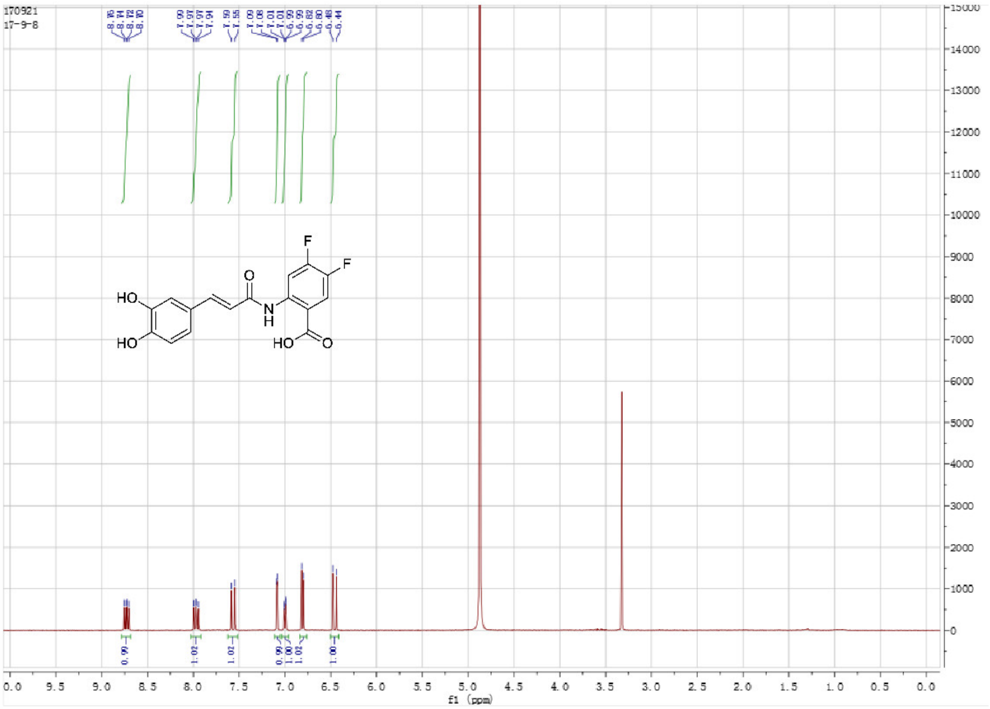


**Figure S3.** $^{1}H$ spectrum of isolated compound **9** in $CD_3OD$.

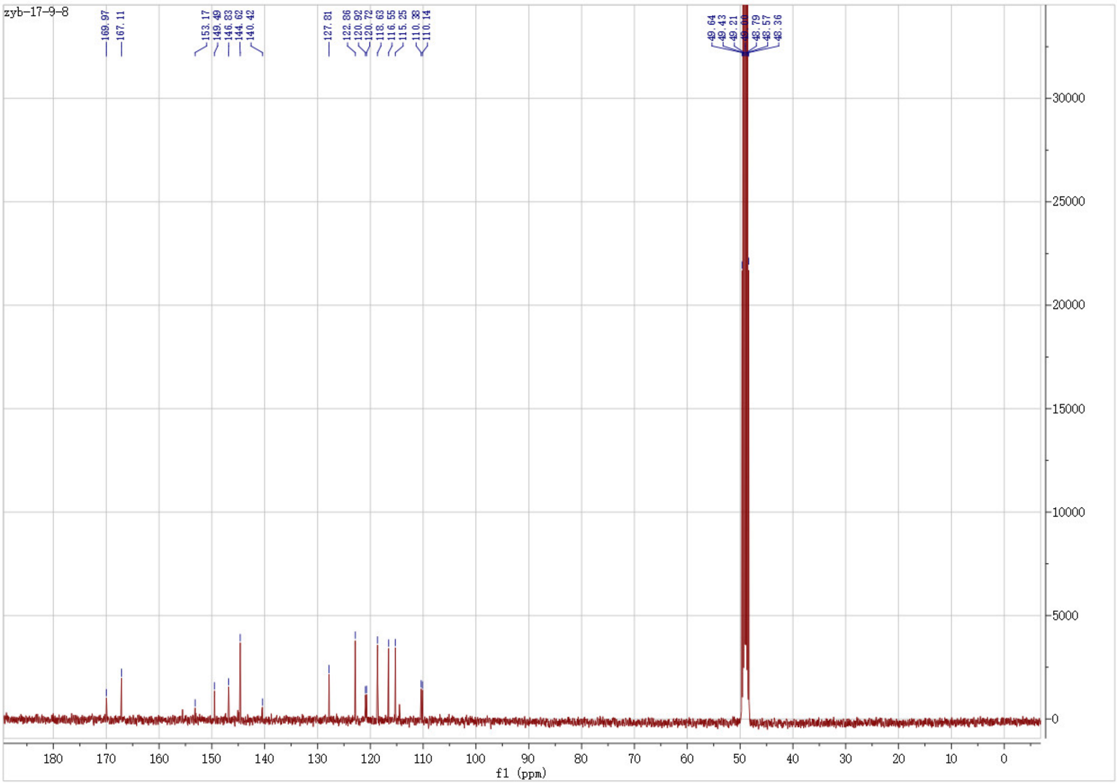


**Figure S4.** $^{13}C$ spectrum of isolated compound **9** in $CD_3OD$.

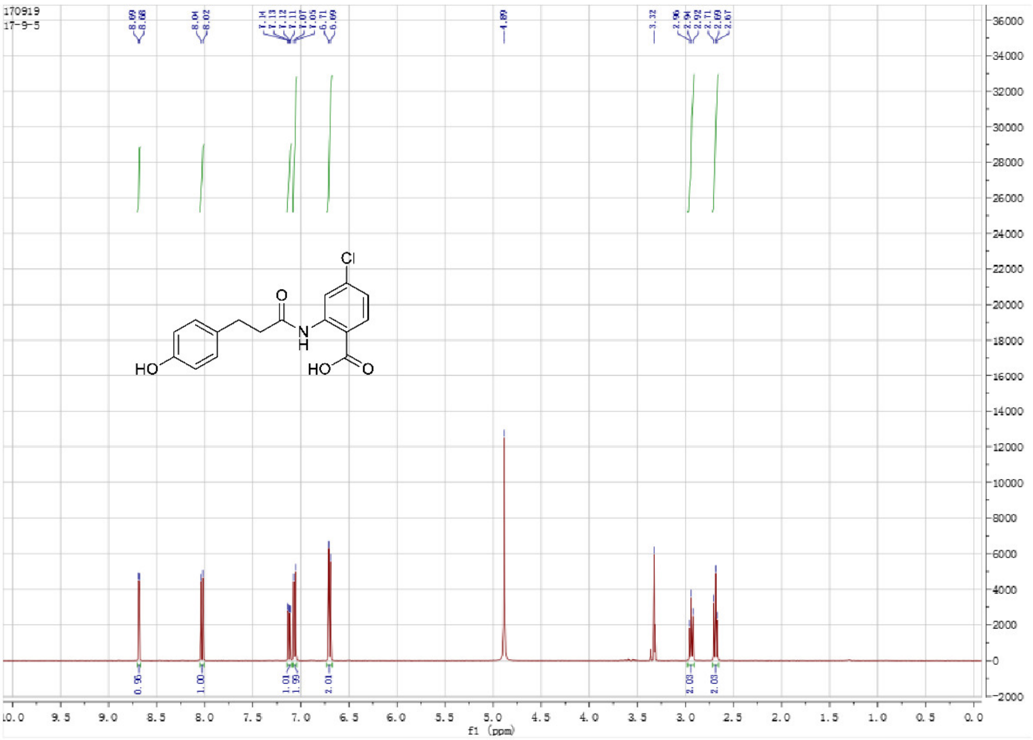

**Figure S5.** $^{1}$H spectrum of isolated compound **10** in $CD_3OD$.

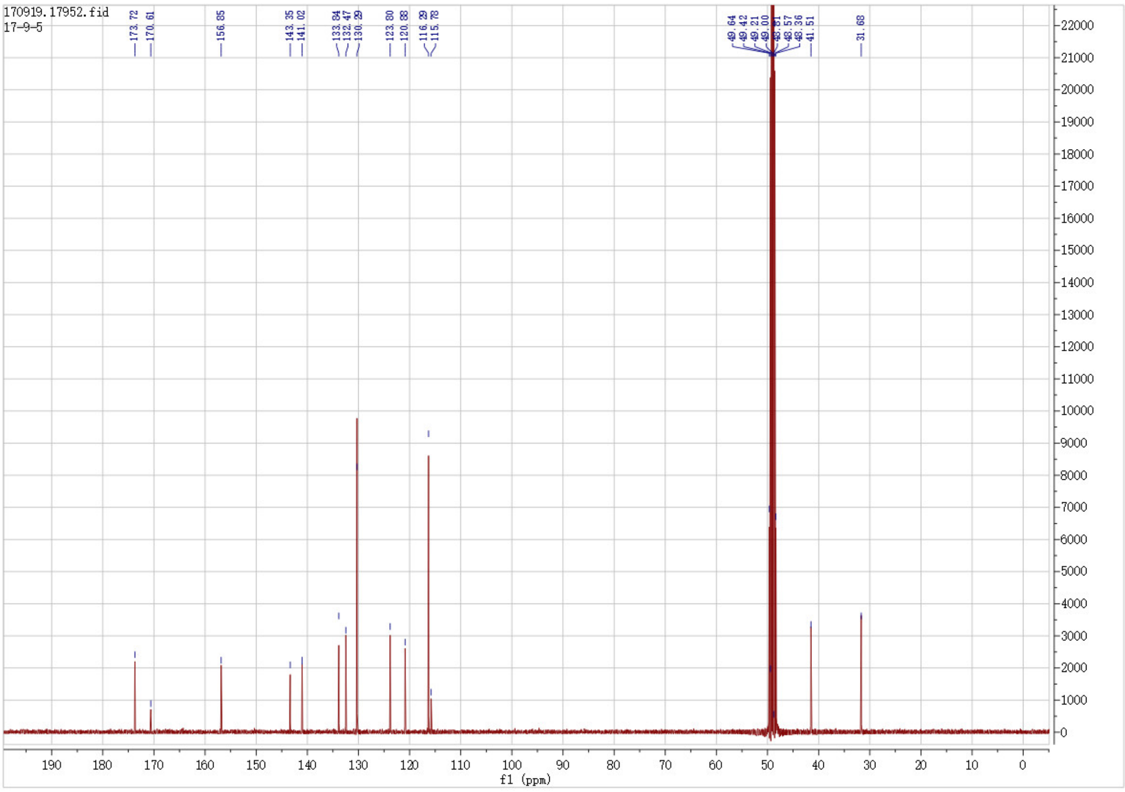

**Figure S6.** $^{13}$C spectrum of isolated compound **10** in $CD_3OD$.

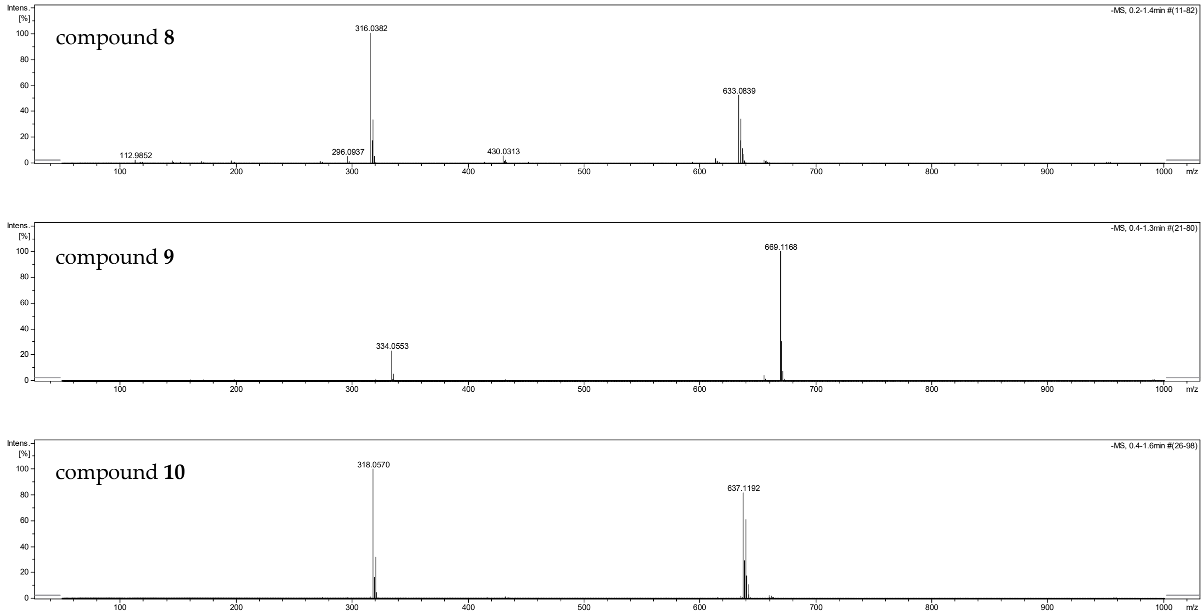


**Figure S7.** MS spectra of compounds **8-10**.
